# The Creation of an Interprofessional Education (IPE) Strategy Utilising a Delphi Method

**DOI:** 10.1111/tct.70098

**Published:** 2025-05-04

**Authors:** Nebras Alghanaim, Samantha Rogers, Gabrielle Finn, Jo Hart

**Affiliations:** ^1^ Division of Medical Education, Faculty of Biology, Medicine and Health University of Manchester Manchester UK; ^2^ Faculty of Medicine King Saud bin Abdulaziz University for Health Sciences Jeddah Saudi Arabia; ^3^ King Abdullah International Medical Research Center Jeddah Saudi Arabia; ^4^ Ministry of the National Guard‐ Health Affairs Jeddah Saudi Arabia; ^5^ Division of Nursing, Midwifery & Social Work, Faculty of Biology, Medicine and Health University of Manchester Manchester UK

**Keywords:** E‐Delphi, collaboration, healthcare, interprofessional education, strategy

## Abstract

**Background:**

Support for interprofessional education (IPE) is growing, with regulatory bodies requiring its inclusion in undergraduate healthcare programmes. Although the IPE Core Competencies and Guidelines offer principles for guiding IPE implementation, they lack practical application guidance. Bridging this gap necessitates tools to translate these frameworks into actionable practices. We developed an IPE strategy to overcome barriers by providing a clear roadmap to IPE implementation.

**Method:**

The study designed a three‐round E‐Delphi. Initially, the research team created 24 strategic statements by consolidating existing IPE competencies. In Round 1, the panel could accept, reject, modify or add new statements. In Round 2, they could accept, reject or modify the revised statements. By Round 3, the panel either accepted or rejected the final statements. The research team analysed the levels of consensus, set at 80% agreement, and thematically analysed the free‐text comments.

**Findings:**

The Delphi panel consisted of 41 participants in Survey 1 and 43 in Surveys 2 and 3. The study began with 24 strategic statements across three priority areas. By the end of the Delphi process, this increased to 28 statements. Study consensus levels ranged from 74.29% to 100%, and participant retention rates were 85.4%, 67.4% and 62.7%, respectively.

**Conclusion:**

The use of the E‐Delphi method demonstrates its value in gathering diverse input, fostering consensus and enhancing the quality and relevance of IPE strategic development by integrating a broad range of perspectives. Further research on the scalability and long‐term effects of this IPE strategy is warranted.

## 1 Background

Collaborative practice within healthcare is widely recognised as a fundamental competency for healthcare professionals across diverse settings. However, poor understanding of team members' roles and unrealistic expectations are common barriers to collaborative practice [1]. The World Health Organization (WHO) advocates interprofessional education (IPE) as a solution. IPE involves learners from different professions learning about, from and with each other to improve collaboration and health outcomes [2]. Benefits include improved care coordination, leadership, conflict resolution, efficient resource use and better healthcare outcomes [2, 3].

*IPE involves learners from different professions learning about, from and with each other to improve collaboration and health outcomes*.

Support for IPE has grown significantly, with regulatory bodies mandating its inclusion in undergraduate healthcare programmes. Yet, several barriers hinder its implementation, including rigid, discipline‐specific curricula, insufficient faculty competence, inadequate infrastructure and resources, limited organisational support, centralised decision‐making and socialisation challenges such as hierarchical power dynamics, stereotyping and disciplinary differences [4]. Addressing these challenges requires targeted efforts in leadership, faculty development, resource allocation, administrative support, curriculum reform and cultural transformation [5]. A recent scoping review on IPE implementation revealed an absence of studies employing a structured consensus approach [4], highlighting the need for a more coordinated strategy that incorporates stakeholder input and aligns with institutional goals for sustainable implementation.

Although faculty development in IPE implementation is widely recognised [6], there is limited evidence to guide this process [7]. This gap is further exacerbated by challenges in translating IPE frameworks into practical applications. For example, although the IPEC Core Competencies for Collaborative Practice [8, 9] and CAIPE's IPE Guidelines [10] provide overarching principles for guiding IPE implementation, they offer limited guidance on practical application. Bridging this gap requires developing valuable tools to transform these frameworks into practical, actionable practices. Strategic planning provides a robust approach to addressing these challenges. A well‐designed IPE strategy may help overcome barriers by providing a clear roadmap and fostering institutional buy‐in.

*A well‐designed IPE strategy may help overcome barriers by providing a clear roadmap and fostering institutional buy‐in*.

An effective IPE strategy requires diverse input to address discipline‐specific needs. Collaboration is key, as it unites varied perspectives, fosters innovation, clarifies roles, reduces conflict [2, 10] and supports quality assurance through continuous feedback and evaluation [11]. It also enhances feasibility via resource sharing, aligns with accreditation standards [8, 9] and promotes sustainability through strong partnerships and adaptability [7].

*An effective IPE strategy requires diverse input to address discipline‐specific needs*.

In our institution, it became clear that implementing IPE Core Competencies alone was insufficient to meet our goals. As a faculty with academic programmes spanning multiple healthcare disciplines, including audiology, biosciences, dentistry, medicine, midwifery, nursing, optometry, pharmacy, psychology, public health and data, social work and speech and language therapy, we recognised the need for a unified and coordinated approach to developing and implementing IPE activities across the faculty. Developing a clearly defined IPE strategy was critical to address this. The strategy needed to align with stakeholder needs by incorporating structured decision‐making processes to gather input and refine decisions. It also required a clear and well‐organised framework to overcome barriers, promote collaboration and deliver meaningful and sustainable outcomes for our healthcare programmes. This study aimed to develop an IPE strategy for effective IPE implementation by utilising the E‐Delphi method to build consensus and foster mutual agreement among diverse stakeholders.

*A clear and well‐organised framework to overcome barriers, promote collaboration and deliver meaningful and sustainable outcomes*.

## 2 Methods

**Research Question:** What key IPE strategies for effective IPE implementation can be identified through stakeholder consensus using the E‐Delphi method?

This study employed a three‐round E‐Delphi method to gather expert opinions through an iterative process, prioritising group consensus over individual perspectives and drawing on shared collegial knowledge [11]. The E‐Delphi method ensures balanced contributions by maintaining anonymity and equal status among participants, preventing dominance by strong personalities [12]. The Delphi method is widely used in health and education research for developing standards and policies [13]. It offers a cost‐effective and efficient approach, enabling participants to contribute flexibly from any location and enhances decision‐making [11].

*E‐Delphi method ensures balanced contributions by maintaining anonymity and equal status among participants, preventing dominance by strong personalities*.

This study was conducted by an interdisciplinary team comprising representatives from medicine, nursing and psychology, all with expertise in healthcare education and Delphi methodology. The study followed DELPHISTAR reporting guidance, ensuring a rigorous, transparent and standardised approach to the Delphi process [14].

### 2.1 The Delphi Process

#### 2.1.1 Initial IPE Strategy Development

The research team developed a baseline strategy, which served as a foundational framework for refinement and further development by the Delphi panel. This initial strategy was informed by core competencies, such as those outlined by the IPE Core Collaborative [8, 9], which defines the essential knowledge, skills and behaviours for effective IPE. In addition, key frameworks and guidelines, including the WHO's Framework for Action on IPE [2], CAIPE's IPE Guidelines [10] and Silver and Leslie's Framework for Faculty Development in Continuing IPE [7], were integrated to embed evidence‐based approaches and best practices. These resources provided valuable guidance for incorporating IPE into curricula and addressed common implementation challenges. To ensure alignment with the University's broader strategic goals of innovation, inclusion and excellence, the strategy focused on three priority areas: (1) connectivity, collaboration and partnership; (2) promoting quality; and (3) stability, sustainability and growth. Strategic objectives supported these priorities, including leadership and governance, communication and networking, training and development, standards and quality assurance, research and evaluation, accessibility, preparation and planning and finance. Each strategic objective had a series of actionable practices. This comprehensive approach aimed to create a robust and sustainable IPE strategy.

### 2.2 Selecting the Panel

Experts are individuals with a high level of knowledge or skill related to a specific subject or activity [15]. Participants were required to have significant knowledge, skills and experience in delivering IPE to undergraduate or postgraduate healthcare students. Prospective panellists were identified through purposive sampling using a ‘snowballing’ technique to ensure adequate representation [16] of specialities across healthcare disciplines.

Educational leaders, such as programme leads, from disciplines including audiology, biosciences, dentistry, medicine, midwifery, nursing, optometry, pharmacy, psychology, public health and data, social work and speech and language Therapy were invited to share information about the research project with experts in IPE. Interested individuals were encouraged to contact the research team directly. Additionally, the team extended invitations to national and international academics and clinical practitioners with IPE expertise. Integrating national and international perspectives into strategy development enhances the process by drawing on diverse knowledge and adopting global best practices. This approach provides broader insights, fosters adaptability and promotes collaborative efforts [17]. In addition, it strengthens cultural competence, fosters innovation and enhances credibility, ensuring the strategy stays proactive and relevant by addressing future trends and challenges [18]. The eligibility criteria were as follows:

- At least 2 years of experience teaching IPE.
- Demonstrated involvement in research related to IPE.
- Held a degree or postgraduate teaching specialising in teaching and learning.

All the above criteria were required for inclusion as a participant.

Although there is no agreed‐upon ideal panel size for the Delphi process, a sample size of 15–30 participants is generally deemed sufficient for most purposes [15].

Undergraduate and postgraduate students were also recruited for this study. Their involvement ensured the strategy was relevant, inclusive and effective by aligning it with their needs, fostering a sense of ownership and empowering them to participate in decision‐making. This collaboration provided practical feedback, encouraged innovation and fostered trust between students and the institution. Strategies co‐created with student input are more likely to succeed, gain widespread acceptance and ensure sustainability, fostering growth and a stronger sense of community [19, 20]. The eligibility criteria were as follows:

- Experience in IPE as a learner or educator.
- Completed research in IPE, such as a master's dissertation or PhD thesis.

Participants were eligible if they met either of these criteria.

### 2.3 Data Collection

This E‐Delphi survey took place between September and December 2023 and was carried out in three rounds. The strategy was formatted into electronic surveys using Qualtrics software [21], providing a user‐friendly platform for data collection. A link to these surveys was emailed to all eligible panel members, ensuring accessibility and streamlined participation in the Delphi process [21]. A participation information sheet was attached to the initial email, inviting expressions of interest and written consent gained before starting the first E‐Delphi Survey.

#### 2.3.1 First E‐Delphi Survey

All identified experts and students were invited by email with a survey link. Participants reviewed strategic priorities and objectives and were given the choice to accept, reject, modify or propose new statements. They were also encouraged to provide qualitative feedback through a free‐text comment box, allowing for more detailed and nuanced input.

#### 2.3.2 Second E‐Delphi Survey

Based on Survey 1 results, strategic priorities and objectives that achieved consensus were retained, whereas others were removed or refined based on participants' comments. In Survey 2, participants reviewed the updated statements, accepting, rejecting or suggesting modifications.

#### 2.3.3 Third E‐Delphi Survey

Following Survey 2 results, strategic priorities and objectives were further revised. Items reaching consensus were retained, whereas others were removed and refinements were made based on feedback. In Survey 3, participants reviewed and accepted or rejected the updated statements. At each round, participants had approximately 3 weeks to complete the survey. After 2 weeks, non‐responders received a reminder.

We adopted the accept, reject, modify approach because it has advantages over the Likert scale in Delphi studies, particularly in achieving actionable consensus. This method requires definitive choices, simplifying data analysis and reducing ambiguity and interpretation bias [22, 23]. Enabling participants to suggest modifications supports the iterative refinement of frameworks or curricula. This approach prioritises actionable outcomes and structured decision‐making, offering a practical way to gather expert insights [13, 23].

### 2.4 Data Analysis

The study established an a priori acceptance consensus threshold of 80% agreement for strategic statements to ensure that only those with strong collective support were retained; statements falling below this benchmark were removed in subsequent rounds [24]. After each round, qualitative comments were analysed using thematic analysis [16] and synthesised to find common themes or points of divergence. This synthesis was used to create or revise statements presented to participants in later rounds for rating or further discussion. Statements that reached consensus but were accompanied by unclear or ambiguous free‐text comments, which did not provide sufficient guidance for further development, were presented again in subsequent rounds. These statements were re‐evaluated in the same format to gather additional input and clarification from the panel, ensuring their refinement and alignment with stakeholder perspectives. To ensure consistent decision‐making, the research team documented how qualitative comments influenced later iterations. This approach helped keep transparency [22] and created an audit trail. These approaches fostered active collaboration, ensuring the strategy was evidence‐based and reflected diverse perspectives. High engagement from the panel is crucial for the success of any Delphi study; we aimed to achieve a response rate above 70% to uphold the study's validity and reliability [25].

### 2.5 Ethical Approval

The study obtained ethical approval from a Proportionate UREC Committee (Reference: 2023‐16610‐30096). In line with the Delphi method, expert contributions to the surveys remained anonymous to the research team [15].

## 3 Findings

A total of 111 participants were invited to the study. The panel included internal (*n* = 14) and external academics (*n* = 16), as well as postgraduate (*n* = 10) and undergraduate (*n* = 3) students with significant IPE experience. Disciplines represented included biosciences, medicine, midwifery, nursing, optometry, pharmacy and speech and language therapy (see Table 1). However, there was no representation from audiology, dentistry, psychology, social work or public health and data.

**TABLE 1:** Demographic characteristics of the Delphi participants.

Demographic characteristics of the Delphi participants		
Country	UK	23
	USA	3
	Canada	2
	Saudi Arabia	10
	Australia	2
	New Zealand	2
	Portugal	1
Participant category	External academics	16
	Internal academics	14
	Postgraduate students	10
	Undergraduate students	3
Professions	Nursing	9
	Midwifery	1
	Medicine	11
	Pharmacy	7
	Speech therapy	2
	Optometry	2
	Occupational therapy	1
	Respiratory therapy	1
	Paramedic	3
	Radiotherapy	1
	Dietitian	1
	Biomedical engineering	1
	Chemistry	1
	Biology	1
	Applied biological anthropology	1

**FIGURE 1:**
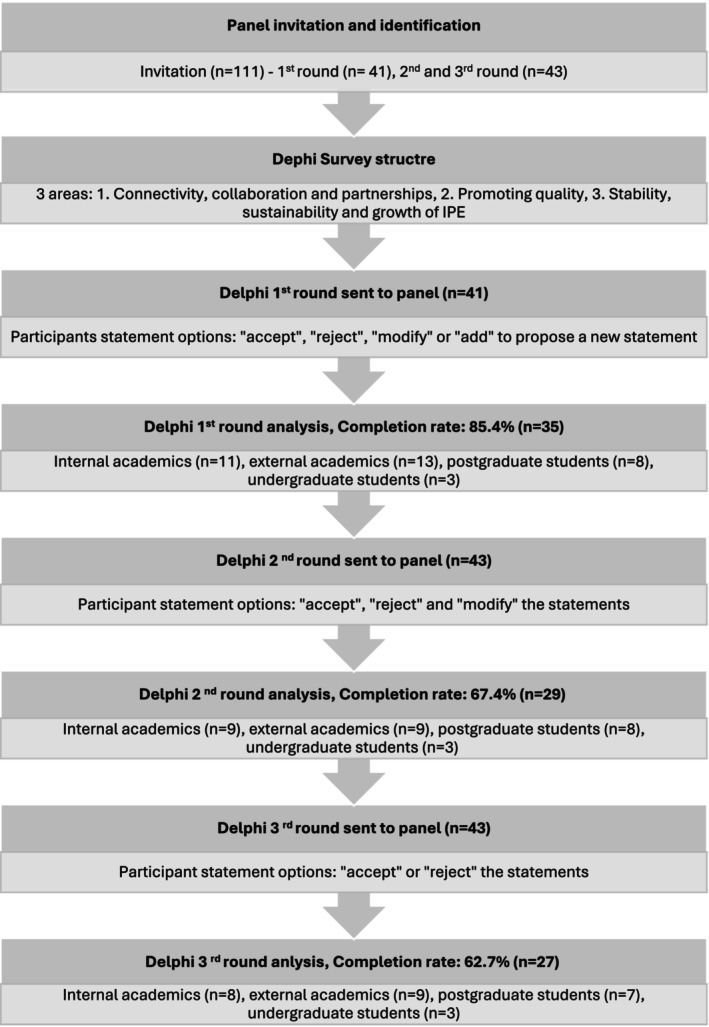
Survey completion rates and demographic composition of the panel members across Delphi rounds.

In Survey 1, 41 responded; Surveys 2 and 3 saw 43 participants, including 2 new additions. The target response rate of 70% per round was achieved in Survey 1 (over 80%) but not sustained in later surveys. Figure 1 outlines response rates by round.

Consensus levels ranged from 74.29% to 100%. Statements below the 80% threshold were removed, whereas those under 100% were revised based on participant feedback. This iterative process enabled the refinement of strategic statements using free‐text insights.

### 3.1 Overview of Strategy Development

At the start of the study, 24 strategy statements were organised under three strategic priorities, further divided into seven strategic objectives. By the end of the Delphi process, the number of strategic statements increased to 28, reflecting the iterative refinement process informed by participant feedback.

#### 3.1.1 Development of Strategic Statements Through Delphi Rounds

- Survey 1: Of the 24 initial statements, 11 were accepted and 12 were revised and included in Round 2. One was removed due to low consensus and five new statements were added.
- Survey 2: Out of the 28 statements (11 accepted statements and 12 revised statement from Round 1 plus 5 new additions), 14 were accepted, and 13 were revised for Round 3. One was removed and one new statement was added.
- Survey 3: All 28 statements were accepted.

Free‐text comments reinforced established themes in the literature while providing new insights for developing an effective IPE strategy. Core themes included collaboration, leadership and community building as essential foundations for success. Participants emphasised the need for evidence‐based, clearly defined curricula with standardised outcomes to ensure consistency and alignment with institutional goals. Faculty support was viewed as crucial, highlighting the importance of structured training, sufficient resources and practical strategies to enhance engagement. Ongoing evaluation was considered essential for ensuring quality and impact, with a focus on distinguishing between routine evaluation and research and sharing findings broadly. Strategic alignment with global standards was considered vital for sustainability and participants emphasised the need for institutional commitment, clear objectives and actionable outcomes. Key recommendations included fostering cross‐school collaboration, appointment of IPE leads, integration of clinical competencies into curricula and strengthening support for research and faculty development. Table 2 shows an example of how free‐text comments were used to refine IPE strategic statements. Table 3 presents the finalised IPE strategy.

**TABLE 2:** Example of how participants free‐text comments were used to refine and develop strategic statements.

	Initial strategic statement generated by the research team	Consensus level and free‐text comments from first Delphi round	Revised strategic statement for second Delphi round	Consensus level and free‐text comments from first Delphi round	Revised strategic statement for second Delphi round
Leadership and governance	B1.1.2 Appoint an Academic Lead for Interprofessional Education to, for example, facilitate understanding, interpretation and implementation of IPE standards, coordinate IPE activities, provide guidance in assessment strategies and identify barriers to progress	**Consensus level**: 82.86% **Free‐text comments**: Develop explicit terms of reference for the academic lead, including procedures for regular input and communication with all stakeholders, to ensure that the role effectively meets the requirements and concerns of all parties concerned I would recommend appointing more than one lead to facilitate and coordinate the activities involved in interprofessional education. I think that would be more efficient and practical	B1.1.2 Appoint an Academic Lead from each healthcare programme for Interprofessional Education to, for example, facilitate understanding, interpretation, and implementation of IPE standards, coordinate IPE activities, provide guidance in assessment strategies and identify barriers to progress within the university. Leadership will be clearly defined, and appropriate governance models and processes will be explicitly described	**Consensus level**: 96.55% **Free‐text comments**: I would recommend appointing more than one lead to facilitate and coordinate the activities involved in interprofessional education. I think that would be more efficient and practical	B1.1.2 Appoint a lead/s from each healthcare programme for Interprofessional Education to, for example, facilitate understanding, interpretation, and implementation of IPE standards, coordinate IPE activities, provide guidance in assessment strategies and identify barriers to progress within the university. Leadership will be clearly defined, and appropriate governance models and processes will be explicitly described

**TABLE 3:** The final interprofessional education (IPE) strategy.

Strategic priority (1): Connectivity, collaboration and partnerships	
Aim:	To establish a shared vision for interprofessional education based on our identity as a learning community within the faculty
We will address this through:	i Leadership and governance: Establishing faculty infrastructure that supports the goals and outcomes of interprofessional education
	ii Communication and networking: Maximising communication and networking opportunities to share best practices and facilitate connectivity, collaboration and partnership between academic staff/faculty members, learners, patients, service users and external stakeholders
i Leadership and governance	
1 Establish an IPE steering committee, with a lead person to lead on developing and implementing the interprofessional education strategy and report progress to the faculty leadership team. Roles and responsibilities will be clearly defined and appropriate governance models and processes will be explicitly described	
2 Appoint a lead/s from each healthcare programme for interprofessional education to, for example, facilitate understanding, interpretation and implementation of IPE standards, coordinate IPE activities, provide guidance in assessment strategies and identify barriers to progress within the university. Leadership will be clearly defined and appropriate governance models and processes will be explicitly described	
3 Ensure interprofessional education is clearly described within the university's strategic plan, with development and delivery supported by the senior leadership team	
4 Appoint student representatives with clearly defined roles and responsibilities to inform the development of interprofessional education	
ii Communications and networking	
5 Establish a community of practice and or a steering committee with clear mechanisms to share best practice, learning and expertise and gain an appreciation of learners' needs across all university healthcare programmes.	
6 Develop a digital platform/virtual learning environment to share resources and expertise, showcase best practices, facilitate collaborations and enhance communications across healthcare programmes within the university.	
7 Continue to develop and establish links with external stakeholders relevant to individual healthcare training requirements, including professional regulatory and statutory bodies, Royal Colleges and NHS (National Health Service) trusts	
Strategic priority (2): Promoting quality	
Aim:	To continue delivering high‐quality IPE that promotes learners' abilities to meet identified learning outcomes/objectives
We will address this through:	Training and development: Providing equitable access to IPE development programmes Standards and quality assurance: Promoting quality‐assured IPE staff training across the professional spectrum Research and evaluation: Fostering evaluation processes that promote a reflective learning culture that leads to improvement
i Training and development	
8 Provide new and existing academic staff/faculty member delivering or with the desire to deliver IPE from all health programmes with flexible and accessible evidence‐based IPE training and development opportunities	
9 Support IPE academic staff/faculty member to participate in advisory committees, professional or practice‐based IPE forums or networks as part of continuing professional development	
10 Develop and implement a road map for professional development designed specifically for IPE academic staff/faculty member from all programmes to discuss desired outcomes and assessment strategies. The professional development plan and/or pathway should include, but not be limited to, membership and engagement with IPE networks, attendance at local/regional/national/international conferences and completion of IPE‐focused study days and/or courses	
ii Standards and quality assurance	
11 Raise awareness and promote the application of regional, national and international IPE competencies/models, for example, but not limited to, IPE Core Competencies (IPEC 2016) and Interprofessional Education Guidelines (CAIPE 2017)	
12 Embed regional, national and international IPE competencies/models/for example, but not limited to, IPE Core Competencies (IPEC 2016) and Interprofessional Education Guidelines (CAIPE 2017), into the design and development of relevant healthcare programmes	
13 Use a periodic review process and feedback to ensure all IPE activities delivered across faculty are feasible, appropriately designed based on programmatic resources and in alignment with the IPE Strategy. This will be measured by quality assurance processes, for example, annual evaluation of IPE activities, incorporating outcomes data, learner, IPE faculty and stakeholders' feedback	
14 Engage in annual peer‐review processes to ensure ongoing development of academic staff/faculty member delivering IPE	
15 Establish a clear process and/or system of reviewing IPE resources, for example, standards of best practice, e‐learning materials, evidence‐based practice, and training and development courses, to ensure staff remain up to date	
iii Research and evaluation	
16 Commit to undertaking evaluations of IPE activity to determine the quality and/or effectiveness of the IPE experience on an individual, divisional, school or faculty level and collaboration with relevant communities, including equality, diversity and inclusion (EDI). Evaluation should include, but not be limited to, feedback from learners, academic staff/faculty member and external stakeholders to use the research and evaluation data for continuous quality improvements	
17 Facilitate appropriate training and supervision for academic staff/faculty member designing and delivering simulation to develop research projects and evaluation processes that consider educational effectiveness and efficiency, patient safety, quality of care and preparedness of the learner for the workforce	
18 Establish systems to actively support and promote the dissemination of outcomes/findings from research and/or evaluation processes in professional/scientific journals, and internal and external conferences	
19 Disseminate evaluation data internally (with proper anonymisation), promoting recognition and improvement at an individual, division, school and faculty level	
Strategic priority (3): Stability, sustainability and growth of interprofessional education	
Aim:	Embrace current and future developments in IPE to enhance the learner's experience
We will address this through:	Accessibility: Improving accessibility, deliverability and utilisation of IPE teaching facilities and equipment; striving for equitable access across healthcare programmes. Preparation and planning: Working together to overcome the challenges associated with IPE Finance: Securing and managing the financial resources to support stability, sustainability and growth of IPE delivery
i Accessibility	
20 Review existing teaching spaces, with a view to developing a system/process for sharing spaces to increase capacity for interprofessional healthcare education and enhance the learner's experience	
21 Identify learning spaces to build and develop IPE learning spaces to deliver interprofessional healthcare education	
22 Ensure digital innovations are accessible for all learners, ensuring an inclusive approach to teaching and learning	
ii Preparation and planning	
23 Assess academic staff/faculty member readiness for IPE growth, for example, knowledge regarding IPE core competencies, IPE guidelines, team communication and learning levels of learners from outside their healthcare programme, using, for example, the Readiness for Interprofessional Learning Scale (RIPLS) Tool	
24 Forecast programme/faculty growth for IPE, including personnel, information technology (IT), E‐learning and librarian support teams, workload, roles and responsibilities, training and development needs and facilities	
25 Identify priorities, areas, benefits, challenges and solutions for IPE within healthcare programmes via a scoping exercise across all healthcare programmes	
26 Undertake curricular mapping across all healthcare programmes to identify potential and appropriate timeframes for delivering IPE activities	
27 Undertaking a scoping exercise to identify healthcare programmes' professional regulatory requirements and aspirations for interprofessional education/practice learning	
iii Finance	
28 Prepare an operational budget considering current and future goals and priorities, including identifying fixed (e.g., maintenance and service contracts), variable (e.g., personnel, training and development for staff, peer review, audit and dissemination of research/scholarly activity) costs, future capital expenditure and human resources	

## 4 Discussion

This study highlights the efficacy of the E‐Delphi method in uniting academics and students from diverse healthcare disciplines to develop a comprehensive and actionable IPE strategy. A key strength of the strategy lies in its inclusion of practical, strategic statements designed to address common barriers to IPE implementation. These include faculty development, resource allocation, administrative support, curriculum reform and cultural transformation.

A core advantage of the strategy is its alignment with established frameworks, including the WHO's *Framework for Action on IPE* [2], CAIPE's *IPE Guidelines* [10], the *IPE Core Competencies* [8, 9] and Silver and Leslie's *Framework for Faculty Development* [7]. These alignments lend the strategy credibility, relevance and effectiveness by building on evidence‐based foundations, promoting consistency and providing structured guidance.

*A core advantage of the strategy is its alignment with established frameworks*.

Preparation and planning were also emphasised. These are areas often overlooked in IPE competencies. The strategy addresses foundational gaps through activities such as faculty readiness assessments, forecasting growth, mapping curricula and aligning with regulatory requirements. This structured approach mitigates unforeseen challenges and supports scalability, adaptability and sustainability across diverse contexts, increasing its long‐term impact [10].

Research and evaluation were prioritised, with built‐in mechanisms for continuous review and refinement. By embedding evidence‐based inquiry and fostering accountability, the strategy remains responsive to evolving best practices. Designed as a replicable framework, it has the potential to support IPE integration at local, national and international levels and is adaptable to various institutional and cultural contexts [11].

The collaborative nature of the E‐Delphi method was central to the success of the strategy. It enabled the transformation of theoretical competencies into actionable practices and fostered a shared vision among diverse stakeholders. These findings support existing research, highlighting the value of stakeholder‐led frameworks in sustaining educational initiatives [26].

A diverse panel representing biosciences, medicine, midwifery, nursing, optometry, pharmacy and speech and language therapy contributes to the development of the strategy. Retention challenges were mitigated through reminders and concise survey design. However, participation declined over time, suggesting the potential value of a hybrid engagement model [12]. Despite the breadth of representation, gaps remained in audiology, dentistry, psychology, social work and public health. The over‐representation of nursing and medicine may have influenced the strategic priorities, whereas the absence of fields such as physiotherapy, radiotherapy and occupational therapy reflected institutional constraints. Addressing these gaps will be crucial to strengthen inclusivity and relevance further.

Consensus levels across the three rounds ranged from 96% to 100%, with iterative refinement enhancing clarity and quality. The independent and collaborative qualitative analysis conducted by the research team contributed to the trustworthiness and validity of the findings [16].

These findings have important implications for the development and implementation of sustainable and impactful IPE strategies. The alignment of free‐text comments with established literature reinforces the validity of the study's outcomes, whereas the emergence of new insights reflects the value of engaging a diverse panel in shaping practical, context‐specific recommendations [27]. The emphasis on collaboration, leadership and community building supports existing evidence that these are foundational to successful IPE initiatives [2]. Furthermore, the call for robust faculty development strategies highlights ongoing challenges in the field and points to concrete areas for institutional investment [2].

*Call for robust faculty development strategies highlights ongoing challenges in the field and points to concrete areas for institutional investment*.

The importance placed on evaluation and strategic alignment with broader standards speaks to a growing need for accountability and adaptability in IPE frameworks [28]. This highlights the necessity for institutions to go beyond surface‐level integration and commit to structural and cultural shifts [29]. Notably, the practical suggestions, such as appointing IPE leads and embedding clinical competencies, offer actionable guidance that can be tailored to various contexts. Overall, these insights enhance the relevance of the study by providing a strategic roadmap informed by both literature and lived experience. They emphasise the crucial role of institutional support in implementing IPE principles in meaningful educational practice [30].

### 4.1 Implications and Future Directions

Despite limitations, including gaps in disciplinary representation, this study achieved strong consensus and produced a framework with significant potential for impact. Future research should actively engage underrepresented disciplines to enhance inclusivity and assess long‐term outcomes. Refining the strategy in light of these insights will reinforce the importance of collaboration, inclusivity, and evidence‐based planning in building sustainable IPE initiatives. The strategy is currently under review by the Vice Dean for Teaching, Learning and Student Experience for faculty‐wide evaluation and approval, marking a significant step toward its large‐scale implementation.

## 5 Conclusion

This study highlights the importance of collaboration and consensus‐building in developing a robust framework for implementing IPE. Through the E‐Delphi method, a strategic framework was co‐created that effectively bridges theoretical constructs and practical applications. The resulting strategy not only aligns with global standards but also provides a sustainable, adaptable model for advancing healthcare education across diverse settings.

## Author Contributions

**Nebras Alghanaim:** writing – original draft, investigation, methodology, software, formal analysis, writing – review and editing, data curation, conceptualization, validation. **Samantha Rogers:** writing – review and editing, methodology, investigation, formal analysis. **Gabrielle Finn:** supervision, writing – review and editing. **Jo Hart:** writing – review and editing, supervision.

## Conflicts of Interest

The authors declare no conflicts of interest.

## Data Availability

The data that support the findings of this study are available on request from the corresponding author. The data are not publicly available due to privacy or ethical restrictions.
